# A Nurse's Charter of Liberty: Viscount Knutsford's Reply to "Ierne."

**Published:** 1918-06-08

**Authors:** 


### THE ECONOMICS OF NURSING.

### A NURSE'S CHARTER OF LIBERTY.

### Viscount Knutsford's Reply to "Ierne."

In your issue of May 25 you print one of "Ierne's" excellent articles under the above title, in which she asks me some questions. By asking them I presume she thinks that my answer will be of some general interest, and so I will reply.

After a kindly reference to the training given to nurses at the London Hospital, she criticises me severely because I set down in my pamphlet "Nursing as a Profession" what I called the "Charter of Liberty" for nurses. It was perhaps a silly name, but I did not mean to attach any importance to the name, and "Ierne" mistakes altogether my intention.

I set out that "Charter" as the *very minimum* of off-duty time, etc., which a nurse should claim. They were not all getting it then, and are not to-day. "Ierne," however, reads it as my ideal of what nurses' hours off duty should be.

I wrote of the "Charter": "I would not advise any young woman to go to any hospital where fewer hours of liberty are given." By "liberty" I meant, of course, "off duty." We have long since given better hours at the "London." The "Charter" insisted on at least two hours off every day in the daylight. When I wrote it a first-rate hospital like the "Middlesex" was giving only two hours every other day, and then not necessarily in the daylight. At the London every nurse and probationer gets three hours off every day, though "Ierne" refers to our daily time off duty as only two in other parts of her article! She is mistaking the hours given at our preliminary training-school for those which obtain at the hospital. The "Charter" insisted on at least three weeks' holiday every year—we give four weeks.

Then "Ierne" asks me "why, above all other workers, nurses should be asked to forfeit their freedom." Who on earth ever asked them to do anything of the sort, unless it be called a "loss of freedom" to agree to serve certain hours and to be bound by that agreement? Every employed woman may be said to "sacrifice her freedom," if it is thought fit to use such ridiculous language, when she enters any service, whether of the State or of an individual, and is bound by certain rules and regulations of that service. But nurses, like every other person, are free to enter the service they select, and free to leave it if they do not like it. My object in writing that pamphlet was to try to get fair terms for nurses, but I notice that anyone who tries to do their best for nurses gets misrepresented. "Ierne" likens me to King John! Poor King John! If she had only known that my name is Canute's-ford she might have compared me to that conceited paddler.

"Ierne" blames me for calling a nurse's life a "hard one" and asks me why it should be hard. My reply is that nursing involves, of necessity, a great deal of the giving of self for the benefit of others, and that is what makes the profession a hard one. It is not the hours of actual work which makes the life hard, but it is the constant keeping the mind "at attention," if I may use such an expression. I suppose some people would think that nursing at a neurasthenic hospital, where the hours are short and where "there is no nursing" (to use a common expression), is light work, yet it is exceptionally exhausting and hard, for the reason I have given makes nursing hard. But I need not pursue this: all who care for nursing will know what I mean, and I am sure "Ierne" knows.

"Ierne," wilfully or not I cannot tell, has woefully misinterpreted my pamphlet, and misunderstood one of my chief desires in hospital work, which has been to improve the conditions of nurses and their treatment generally.—Yours faithfully,

KNUTSFORD.

